# Clinical relevance of a multiorgan focused clinical ultrasound in internal medicine

**DOI:** 10.1186/s13089-022-00269-3

**Published:** 2022-05-12

**Authors:** Ximena Cid-Serra, Alistair Royse, David Canty, Colin Royse

**Affiliations:** 1 Department of Surgery, The University of Melbourne, Melbourne, Australia; 2 Department of Medicine and Community Care, The Royal Melbourne Hospital, Melbourne, Australia; 3 Department of Surgery, The Royal Melbourne Hospital, Melbourne, Australia; 4 Department of Anesthesia and Pain Management, The Royal Melbourne Hospital, Melbourne, Australia; 5 Department of Medicine, Monash University, Melbourne, Australia; 6 Department of Anesthesia and Perioperative Medicine, Monash Health, Melbourne, Australia; 7 Department of Anaesthesia and Pain Management, The Royal Melbourne Hospital, Melbourne, Australia; 8 Australian Director of Outcomes Research Consortium, Cleveland, The Clinic, Cleveland, USA

**Dear Editor,**

In December 2021, our group published in JAMA Network Open the main result of a randomized controlled trial (RCT) assessing the impact of a multiorgan focused clinical ultrasound (FCU) on the length of hospital stay of patients admitted to internal medicine units with cardiopulmonary presentations. FCU involved cardiac, lung and proximal lower limb veins ultrasound performed in the first 24 h of admission. We did not find a statistically significant difference in the hospital length of stay between groups [1]. Early this year, Luigi Vetrugno et al. published a *letter to the editor* analyzing whether based on our results, FCU would be considered a positive, neutral, or harmful tool [2]. The aim of our letter is to provide further information for a comprehensive answer to that question.

In addition to the outcomes reported, we assessed the effect of FCU on the clinical decision-making process using a clinical assessment form before and immediately after the FCU findings were revealed to the treating team [3]. This information was gathered only from patients allocated to the intervention group (*n* = 124). After knowing the FCU findings, the treating physician changed their assessment of the patient's hemodynamic state in 63 (51%) participants and modified the primary diagnosis in 34 (2*7*%) and was able to rule out the second most likely diagnosis in 29 (23%) of them. Findings suggesting left ventricle (LV) diastolic failure with preserved systolic function was found in 30 (24%) participants. The proportion of findings that were previously unknown by the treating team are illustrated in Fig. 1. After FCU, the treating team: added or removed pharmacological treatment in 30 (24%) participants; modified the requirement of imaging tests in 64 (52%) and blood tests in 57 (46%) of the participants; and changed the option of consulting another specialist in 21 (17%) of them. Details on the direction of the management changes (“step-down” versus “step-up”) are summarized in Fig. 2.

**Fig. 1:**
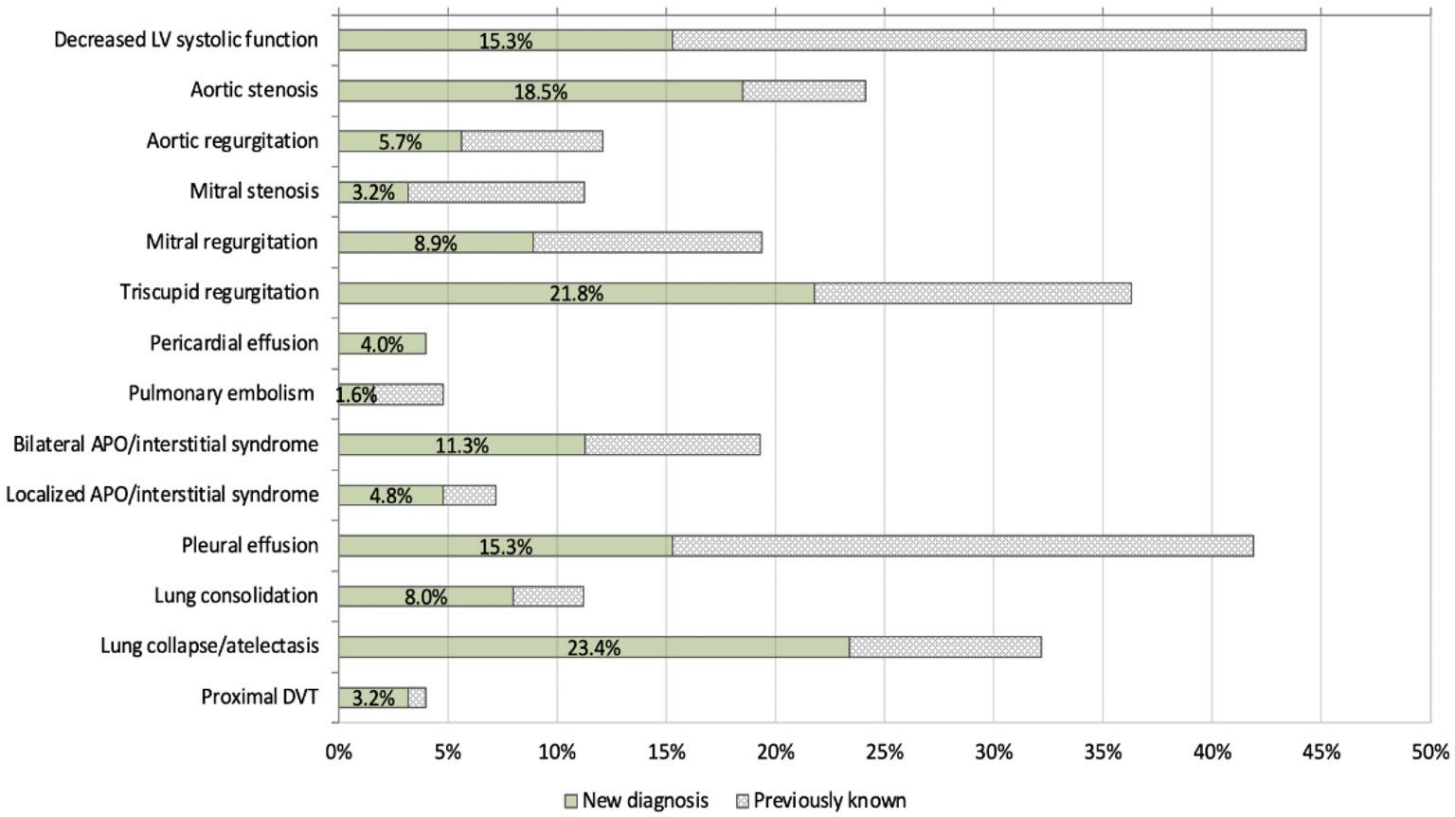
Proportion of previously unknown ultrasound abnormalities. In green (new diagnosis), the proportion of patients in whom FCU identified a pathology that it was unknown and not suspected by the treating team. In gray, the FCU abnormal findings that were already known to the clinical team. *APO* acute pulmonary oedema, *DVT* deep vein thrombosis, *LV* left ventricle

**Fig. 2:**
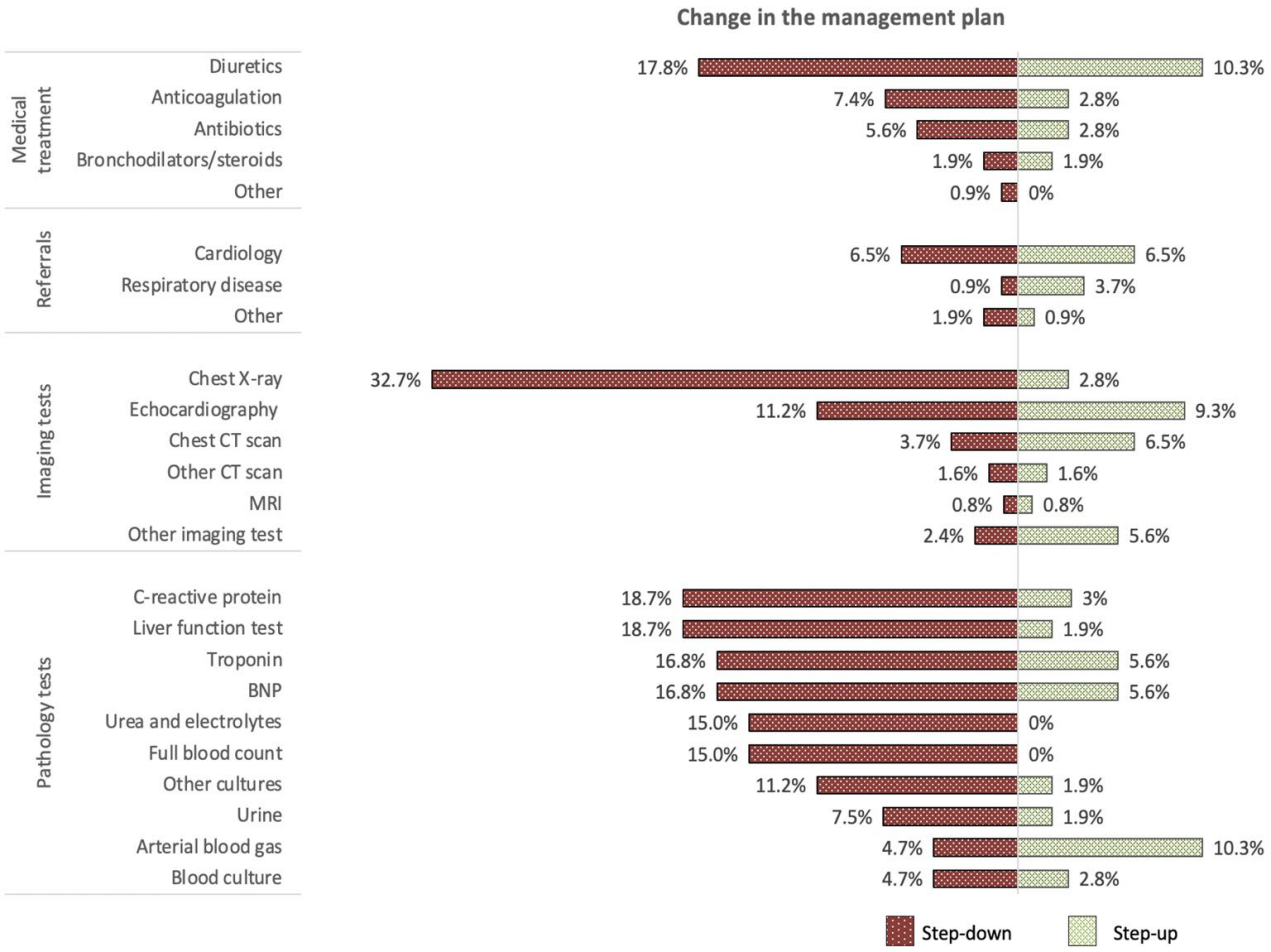
Change in management after FCU. The proportion of participants in the intervention group who had a modification in the medical plan after FCU. The left side of the graph (red) illustrates the percentage of participants who had a step-down in management after FCU and the right side (green) who had a step-up. “Step-down” implies that medical therapies were stopped or referrals, imaging tests, and pathology tests were not requested compared to the pre-FCU plan. “Step up” infers the opposite. *CT* computed tomography, *MRI* magnetic resonance imaging, *BNP* brain natriuretic peptide

Overall, we agree with Vetrugno et al. [2] that the main utility of FCU is to improve physicians’ clinical performance, which is comparable to the role of the stethoscope or the pulmonary artery catheter. Therefore, we adhere to the call to be cautious in defining the clinical relevance of FCU based solely on its impact (or not) on clinical outcomes. In our study, despite not reducing the length of hospital stay, FCU assisted with the clinician’s diagnostic formulation, the decision of requesting further investigations and commencing or ceasing medical therapies.

Its role in supporting the clinical decision-making process could be considered sufficient to call FCU a *positiv*e tool. However, we genuinely believe that FCU can potentially lead to earlier hospital discharge in some specific clinical scenarios. Furthermore, this effect is probably enhanced when FCU is used repeatedly during the hospital stay to guide therapy and not merely for the initial diagnostic assessment.

## Data Availability

Deidentified data available for researchers whose proposed use of the data has been approved and after a proposal and signed data access agreement.

## References

[1] Cid-Serra X, Royse A, Canty D, Johnson DF, Maier AB, Fazio T (2021). Effect of a multiorgan focused clinical ultrasonography on length of stay in patients admitted with a cardiopulmonary diagnosis: a randomized clinical trial. JAMA Netw Open.

[2] Vetrugno L, Ventin M, Maggiore SM (2022). Focus clinical ultrasonography: again competency differs from the patient outcome. Ultrasound J.

[3] Cid X, Canty D, Royse A, Maier AB, Johnson D, El-Ansary D (2020). Impact of point-of-care ultrasound on the hospital length of stay for internal medicine inpatients with cardiopulmonary diagnosis at admission: study protocol of a randomized controlled trial-the IMFCU-1 (internal medicine focused clinical ultrasound) study. Trials.

